# Correlates of the molecular vaginal microbiota composition of African women

**DOI:** 10.1186/s12879-015-0831-1

**Published:** 2015-02-21

**Authors:** Raju Gautam, Hanneke Borgdorff, Vicky Jespers, Suzanna C Francis, Rita Verhelst, Mary Mwaura, Sinead Delany-Moretlwe, Gilles Ndayisaba, Jordan K Kyongo, Liselotte Hardy, Joris Menten, Tania Crucitti, Evgeni Tsivtsivadze, Frank Schuren, Janneke HHM van de Wijgert, for the Vaginal Biomarkers Study Group

**Affiliations:** Institute of Infection and Global Health, University of Liverpool, Liverpool, UK; Amsterdam Institute for Global Health and Development and Academic Medical Center, Amsterdam, The Netherlands; Institute of Tropical Medicine, Antwerp, Belgium; London School for Hygiene and Tropical Medicine, MRC Tropical Epidemiology Group, London, UK; Ghent University, International Centre for Reproductive Health, Ghent, Belgium; International Centre for Reproductive Health Kenya, Mombasa, Kenya; Wits Reproductive Health and HIV Research Institute, Witwatersrand University, Johannesburg, South Africa; Rinda Ubuzima, Kigali, Rwanda; TNO Quality of Life, Zeist, The Netherlands; Department of Clinical Infection, Immunology and Microbiology, Institute of Infection and Global Health, University of Liverpool, Ronald Ross Building, West Derby Street, Liverpool, L69 7BE UK

**Keywords:** Bacterial vaginosis, Vaginal microbiome, Vaginal microbiota, *Lactobacillus*, Candidiasis, Sexually transmitted infections, Urinary tract infections, Women, HIV, Africa

## Abstract

**Background:**

Sociodemographic, behavioral and clinical correlates of the vaginal microbiome (VMB) as characterized by molecular methods have not been adequately studied. VMB dominated by bacteria other than lactobacilli may cause inflammation, which may facilitate HIV acquisition and other adverse reproductive health outcomes.

**Methods:**

We characterized the VMB of women in Kenya, Rwanda, South Africa and Tanzania (KRST) using a 16S rDNA phylogenetic microarray. Cytokines were quantified in cervicovaginal lavages. Potential sociodemographic, behavioral, and clinical correlates were also evaluated.

**Results:**

Three hundred thirteen samples from 230 women were available for analysis. Five VMB clusters were identified: one cluster each dominated by *Lactobacillus crispatus* (KRST-I) and *L. iners* (KRST-II), and three clusters not dominated by a single species but containing multiple (facultative) anaerobes (KRST-III/IV/V). Women in clusters KRST-I and II had lower mean concentrations of interleukin (IL)-1α (p < 0.001) and Granulocyte Colony Stimulating Factor (G-CSF) (p = 0.01), but higher concentrations of interferon-γ-induced protein (IP-10) (p < 0.01) than women in clusters KRST-III/IV/V. A lower proportion of women in cluster KRST-I tested positive for bacterial sexually transmitted infections (STIs; p_trend_ = 0.07) and urinary tract infection (UTI; p = 0.06), and a higher proportion of women in clusters KRST-I and II had vaginal candidiasis (p_trend_ = 0.09), but these associations did not reach statistical significance. Women who reported unusual vaginal discharge were more likely to belong to clusters KRST-III/IV/V (p = 0.05).

**Conclusion:**

Vaginal dysbiosis in African women was significantly associated with vaginal inflammation; the associations with increased prevalence of STIs and UTI, and decreased prevalence of vaginal candidiasis, should be confirmed in larger studies.

**Electronic supplementary material:**

The online version of this article (doi:10.1186/s12879-015-0831-1) contains supplementary material, which is available to authorized users.

## Background

Lactobacilli-dominated vaginal microbiota (VMB) have traditionally been considered to promote reproductive health of women and their fetuses by maintaining a low vaginal pH (<4.5), which restricts the growth of other bacteria and yeasts [1]. The clinical conditions caused by an imbalanced VMB include bacterial vaginosis (BV) and bacterial vaginitis [2]. In addition, VMB-associated bacterial communities may be influenced by other micro-organisms in the vagina, such as *Candida* species, *Trichomonas vaginalis*, and other sexually transmitted pathogens [3-6].

BV has traditionally been characterized as a reduction of vaginal lactobacilli and an overgrowth of other (facultative) anaerobic bacteria. In clinical settings, BV is typically diagnosed using Amsel criteria (three of the following 4 criteria should be present: 1) clue cells on wet mount microscopy; 2) a ‘fishy’ odour after adding 10% KOH to vaginal secretions; 3) vaginal pH > 4.5; and 4) thin, homogenous vaginal discharge [2]. In 1991, Nugent and colleagues developed a method that could be repeated by scoring a Gram stained slide based on microscopic visualization of three bacterial morphotypes (a Nugent score of 0–3 is considered normal vaginal microbiota, 4–6 intermediate microbiota, and 7–10 BV-positive) [7]. Nugent scoring is considered the gold standard for BV diagnosis and is typically used in research settings.

Since 2002, an increasing number of VMB studies have used molecular characterization of vaginal bacterial communities, such as next generation sequencing, quantitative PCR or microarray analysis of bacterial 16S rRNA genes. A recent review of 63 molecular VMB studies conducted between 2008 and 2013 concluded that lactobacilli-dominated VMB are indeed associated with a healthy vaginal micro-environment (but that *Lactobacillus crispatus* is more beneficial than *L. iners*) and that BV is best described as a polybacterial dysbiosis [6]. In most studies, the extent of dysbiosis correlated well with Nugent score and vaginal pH but not with the other Amsel criteria [3,6]. Some studies reported systematic VMB differences across ethnic groups, with Black and Hispanic American women being less likely to have a VMB dominated by *L. crispatus*, and having a higher average vaginal pH, than White and Asian American women [8-10]. However, data on VMB associations with genital immune responses and other potential sociodemographic, behavioral and clinical correlates are scarce and inconsistent [6].

The vaginal micro-environment is also important in the context of vaginal product development, such as vaginal microbicides for HIV prevention. Candidate products should not disturb, and would ideally promote, a lactobacilli-dominated VMB, and should not induce vaginal inflammation [11,12]. In vaginal microbicide safety trials, the VMB has traditionally been assessed by Nugent scoring, and vaginal inflammation by visual inspection during pelvic examination with or without colposcopy and quantification of a select number of cytokines in cervicovaginal lavages (CVLs) [12-15]. However, these data have always been difficult to interpret due to insufficient understanding of the normal background variation in women of different ages, behaviors and clinical conditions.

Here, we describe the bacterial composition of the VMB of different groups of women in four African countries (Kenya, Rwanda, South Africa and Tanzania) using traditional VMB characterization methods (Amsel criteria and Nugent scoring) as well as molecular methods (16S rDNA phylogenetic microarray). Potential VMB correlates, including cytokine concentrations in CVLs, and sociodemographic, behavioral, and clinical characteristics were also evaluated.

## Methods

### Study design and ethical approvals

Samples for phylogenetic microarray testing as well as data on potential VMB correlates were used from two studies [16,17]. The study contributing most samples and data was a multi-country prospective observational cohort study aimed at characterizing novel safety biomarkers for vaginal HIV microbicide development in East and South Africa (referred to as the Vaginal Biomarkers Study). The study was conducted in 2011–2012 with a cohort of 430 women from three African countries (Kenya, Rwanda and South Africa; Additional file 1). The participants included 109 HIV-negative adult women each in Kenya and South Africa, 30 HIV-negative adolescent women each in Kenya and South Africa, 30 HIV-negative pregnant women each in Kenya and South Africa, 31 HIV-negative women using traditional vaginal practice in South Africa, 30 HIV-negative adult women at high risk for HIV (mostly female sex workers) in Rwanda, and 30 HIV-positive women in Rwanda.

The second study was an intensive longitudinal study cohort of women conducted in 2009 for evaluating the impact of traditional vaginal practices on the vaginal micro-environment in Northwest Tanzania (referred to as the Tanzania Study; Additional file 1). Study participants were 100 women working in bars, guest-houses and other food and recreational facilities located in three towns adjacent to large gold or diamond mines [17,18].

Both studies were approved by all relevant institutional and national ethics committees (Additional file 1). All participants (or their guardians in the case of minors) provided written informed consent and received a modest reimbursement for each study visit (Additional file 1).

### Study procedures

In the Vaginal Biomarkers Study, women were screened, and eligible consenting women were enrolled within four days of the last day of their menstrual period (visit 1). Most study groups described above included healthy, non-pregnant, HIV-negative women between 16 and 35 years of age. The pregnant women group included women who were at most 14 weeks into gestation as determined by abdominal ultrasound, and the HIV-positive women group consisted of women on antiretroviral treatment (ART) for at least six months, currently asymptomatic and with a CD4 count above 350 cells/μl. Women were excluded from all groups if they had a history of hysterectomy or other genital tract surgery in the three months before the screening visit; never had had penetrative vaginal intercourse; were enrolled in an HIV prevention study involving investigational products; had internal and/or external genital warts at screening and/or enrollment; or were breastfeeding and less than six months post-partum at the time of enrollment. Once enrolled, women returned for six follow-up visits (visits 2 to 5 at biweekly intervals over two menstrual cycles, and visits 6 and 7 at three and six months after visit 5), but this paper focuses on the screening and enrollment (visit 1) visits. The median time between these visits was 25 days (interquartile range (IQR) 14 – 39 days). At screening, women underwent face-to-face interviews, blood and urine sample collection with real-time HIV, pregnancy and urinary tract infection (UTI) testing, a pelvic examination with sample collection, and a general physical exam. Samples were subsequently tested for several sexually transmitted infections (STIs; see below), UTIs, BV by Amsel and Nugent criteria, and vaginal candidiasis. At enrollment, additional samples were collected for microarray testing and immunological assessments (see below).

In the Tanzania Study, participants were enrolled at any time during their menstrual cycle and followed every two to three days for four weeks (12 visits in total). For the microarray testing, two pairs of samples from 20 women (two to four weeks apart) were selected. At each visit women underwent a face-to-face interview, physical examination, pelvic examination, and sample collection for STI and BV by Nugent scoring. Samples for microarray analysis were collected either at enrollment (8 women) or visit 6 (12 women).

Participants in both studies received counseling and condoms free of charge. Women who tested positive for curable STIs, UTI, or symptomatic BV or vaginal candidiasis were treated by study clinicians using national treatment guidelines. All HIV-positive and pregnant women were linked to appropriate care in local public clinics.

### Diagnostic and immunological testing

Cervicovaginal samples were collected in the following order: vaginal pH measurement, vaginal samples, CVLs, and endocervical specimens. All diagnostic tests were conducted at the study sites in Africa, and similar tests were used in the two studies, unless otherwise stated (see Additional file 1 for diagnostic details). HIV status was determined by locally approved rapid testing algorithm. Plasma samples were tested for herpes simplex type 2 (HSV-2) antibodies and for syphilis by Rapid Plasma Reagin test with confirmation by a *Treponema pallidum*-specific test. Endocervical swabs were tested for *Neisseria gonorrhoeae* and *Chlamydia trachomatis* by PCR. Vaginal swabs were used to prepare a wet mount (detection of trichomonads and clue cells, and after KOH addition yeasts and amine smell), a Gram stain for Nugent scoring (done centrally at the Institute of Tropical Medicine in Antwerp), and to inoculate *Trichomonas vaginalis* cultures. UTIs were diagnosed by the presence of white blood cells on a urine dipstick test and pregnancy by urine hCG test. Vaginal pH was measured using pH paper strips (pH range 3.6-6.1).

CVLs were obtained by irrigating the cervix and lateral vaginal walls with 10 ml of sterile normal saline (5 ml of saline in Tanzania), immediately stored at 4–8°C, and processed within two hours of collection. They were centrifuged for 10 minutes at 1,000 rpm (3,500 rpm in Tanzania), and the resulting supernatant and pellet were stored separately at −80°C until shipment. Soluble markers of inflammation in CVLs were quantified by Bio-Plex (Bio-Rad Laboratories NV-SA, Nazareth, Belgium) or ELISA at the ITM in Antwerp in the Vaginal Biomarkers Study [19], and by an in house multiplex bead immunoassay at St. Georges University in London in the Tanzania Study as described previously [20,21].

### Samples for microarray testing

Two sterile Copan vaginal swabs were collected per participant per visit. Copan vaginal swabs were shipped frozen to the ITM in Antwerp. Upon arrival, each swab tip was thawed at room temperature for 30 minutes, 1200 μl of diluted PBS (1 PBS: 9 saline, pH 7.4) was added, and the sample was vortexed for 15 seconds. An aliquot of 600 μl was sent on dry ice to TNO (Zeist, the Netherlands) for phylogenetic microarray analysis. We could not test all available samples by microarray due to funding constraints. In the Vaginal Biomarkers Study, only the samples from 216 women that were available in October 2011 were analyzed. These 216 women each contributed one enrollment sample, 34 women also contributed 61 follow-up samples, and two samples had missing clinical data. Tanzania Study participants contributed 20 enrollment and 20 follow-up samples. After excluding six poor quality samples, the total sample size was 313 samples from 232 women. All 313 samples were used for phylogenetic clustering and ecological analyses, but only one enrollment sample per woman with clinical data (N = 230) was used for all other analyses.

### Microarray testing

The phylogenetic microarray (V-Chip, TNO, Zeist, The Netherlands) has been described previously [3,22]. Briefly, it contained 283 DNA hybridization probes that generated a consistent signal with a signal/background (S/B) ratio > 5, of which 74 16S probes were species-specific, 60 16S probes targeted multiple bacterial species within one genus, 42 16S probes were specific at family or order level, 85 targeted higher taxonomic levels, five were groEL probes, 14 were 18S probes, and three were viral probes. We focused our clustering analyses on these 283 probes, and all additional analyses on the 134 16S probes generating species or genus-specific signals. A probe targeting a bacterium classified by the Ribosomal Database Project as an uncultured bacterium in the *Lachnospiraceae* family matched perfectly with a bacterium recently named BV-associated bacterium 1 (BVAB1) in Genbank (Genbank entry AY724739.1) [23]. We refer to it as BVAB1 and included it in the 134 species/genus-specific probes.

Microarray sample preparation and labeling, amplification and hybridization were described elsewhere [9,22]. Imagene 5.6 software (BioDiscovery, Marina del Rey, USA) was used to read the scanned results and quantify the signal (S) and the background (B). Ratios for S and B were calculated and if S was not confidently above B (S > B + 2*standard deviation (SD) of B), the S/B ratio was set to 1. Slide normalization was performed by Lowess smoothing [24]. We used normalized S/B ratios to estimate bacterial loads, referred to from here onwards as ‘abundance’.

### Statistical analysis

Clustering analysis was performed using Python 2.7.2 version (Python Software Foundation, https://www.python.org/download/releases/2.7.2/). Other statistical analyses were performed using R 3.0.2 version (R Development Core Team, 2013), MATLAB (R2012a, The Math Works, Natick, USA), STATA release 12 (StataCorp, TX, USA) and MS Excel (Microsoft, Washington, USA).

Neighborhood co-regularized multi-view spectral clustering of normalized S/B ratios was used to identify VMB clusters as described before [3,25]. These clusters were named KRST-I to KRST-V (with KRST denoting Kenya, Rwanda, South Africa, and Tanzania). For each sample, the probability of belonging to a particular cluster was calculated by probability decomposition of the co-occurrence matrix. A cut-off probability of 70% was used to assign samples to a cluster. For each cluster, the following microbial ecology parameters were computed: richness (median number of genera), evenness (expressed as a community organization value (Co-value) with 0 representing complete evenness and 100 complete unevenness [26], and the Shannon diversity index [27]. We focused the evenness calculations on the five most abundant bacteria in each cluster to reduce the influence of the long tail of minority species [26]. To compare cumulative Co-values per cluster, an average sample per cluster was generated by calculating median S/B ratios per genus across the samples in that cluster.

To assess associations between VMB clusters and potential correlates, we only included one sample per woman with clinical data (N = 230) and excluded women who could not be assigned to a cluster with at least 70% probability (N = 22). To improve statistical power, the three dysbiotic clusters (KRST-III, KRST-IV and KRST-V) were pooled, and the pooled cluster is referred to as KRST-pIII-V (p for pooled). In regression models, KRST-II and KRST-pIII-V were each compared to KRST-I. VMB correlates were grouped into three groups: 1) sociodemographics, sexual behavior and reproductive history; 2) self-reported symptoms, clinician-observed findings and antibiotic use; and 3) cervicovaginal immunology. Unadjusted associations were assessed by one way ANOVA for continuous variables and Fisher’s exact test for categorical variables; p values were adjusted for false discovery using the linear step up Benjamini-Hochberg procedure, assuming a false discovery rate q = 0.1 and a significance level α = 0.1. An adjusted p value of 0.01 (α = 0.1 * q = 0.1) was considered statistically significant. Adjusted associations were assessed by multinomial logistic regression models with stepwise backward elimination using p ≤ 0.2 as the cut-off in unadjusted models. The final model was selected based on the smallest Akaike Information Criteria (AIC). Microarray testing was done in two batches but we found no evidence for a batch effect in our analyses.

## Results

### VMB clusters

We identified five VMB clusters, which are visualized in a co-occurrence matrix (Additional file 1: Figure S1A-C). Thirty-five samples from 22 women had a probability of <70% belonging to one of the five clusters (Additional file 1: Figure S1A). These samples did not cluster together, and the sociodemographic, behavioral and clinical characteristics of the 22 women did not differ significantly from those of the 208 women who were assigned to a cluster (Additional file 1: Tables S1 and S2).

**Figure 1:**
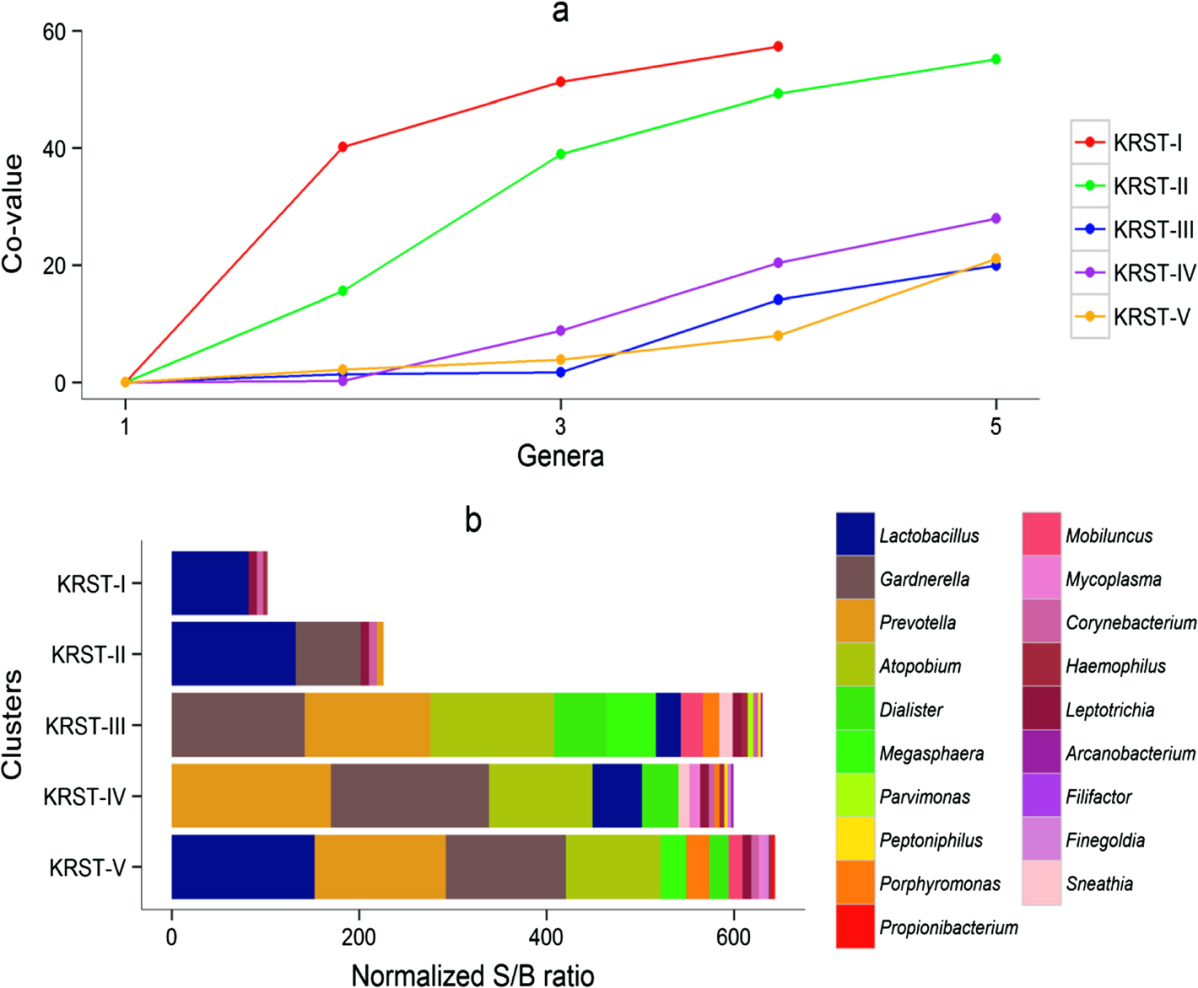
**Median abundance and evenness per cluster. (a)** For each median sample per cluster, the cumulative co-values for the five most abundant genera in descending order are plotted. In **(b)** the actual genera present in each cluster are shown; this clearly illustrates the dominance of lactobacilli in clusters KRST-I and II, and a more even distribution of the other 18 most abundant anaerobic genera in other clusters. Cluster KRST-II also contained *Atopobium vaginae* and *Prevotella* spp., but these are not visible in **(b)** because their abundance in most samples was low. They are visible in the heatmap in Figure 2.

**Figure 2:**
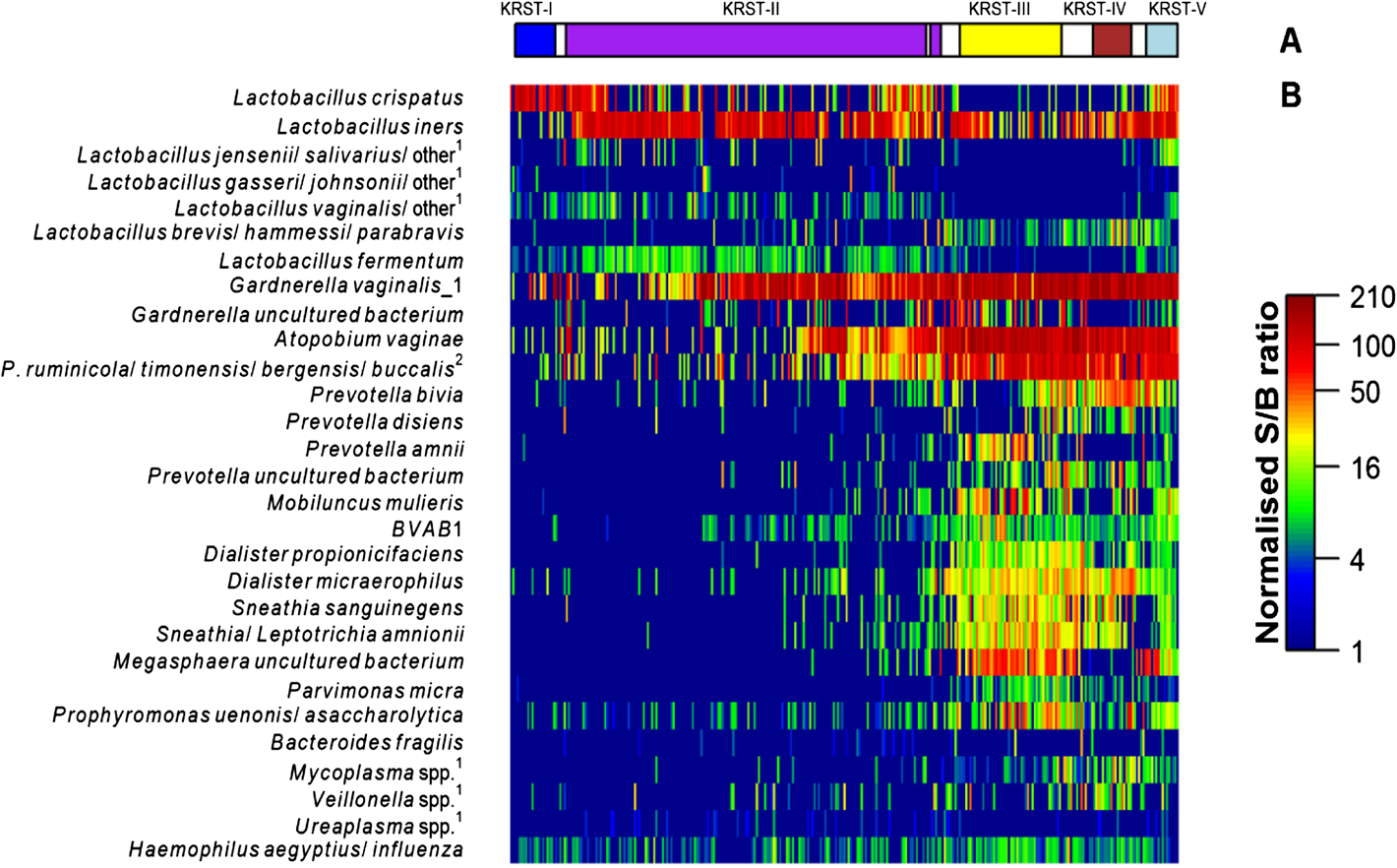
**Bacterial composition of the cervicovaginal microbiome clusters. (a)** shows the five clusters identified by Neighborhood Co-regularized Multi-view Spectral Clustering of microarray data. The white space in between clusters represents samples with less than 70% probability of belonging to a cluster. **(b)** Heatmap, showing normalized S/B ratios of 33 bacterial species/genera, including the most abundant species/genera per cluster and those traditionally known to be associated with BV and frequently reported in literature. ^1^Abbreviated probe name: additional targeted species in the same genus are provided in supplementary information of previously published work [3]. ^2^Abbreviated for *Prevotella*.

The VMB clusters were characterized using ecological parameters (below), Co-values (Figure 1), and a heatmap of S/B ratios of bacteria that were most abundant in this study or have been reported as important in previous studies [3] (Figure 2). Cluster KRST-I was dominated by *L. crispatus* and did not contain other bacterial taxa in high abundance. Only 19 women (9%) were assigned to this cluster. Cluster KRST-II was dominated by *L. iners*, but some samples also had high abundance of *Gardnerella vaginalis*, *Atopobium vaginae*, and *Prevotella* spp (Figure 2). Cluster KRST-II included the majority of women (n = 136, 65.4%). Clusters KRST-III, IV and V each contained multiple anaerobic species in high abundance (most notably *G. vaginalis*, *A. vaginae*, and *Prevotella* spp.) but in different proportions, and a lower abundance of lactobacilli than clusters KRST-I and II. About a quarter (25.5%) of the women were assigned to the combined cluster KRST-pIII-V. Cluster KRST-III had the highest abundance of *Dialister*, *Megasphaera* spp. and *Mobiluncus* spp. and the lowest abundance of *L. iners*. Cluster KRST-IV had the highest abundance of *Prevotella spp*. and the lowest abundance of BVAB1 and *Megasphaera* spp. Cluster KRST-V contained a higher abundance of *L. iners* and other *Lactobacillus* spp. than clusters KRST-III and IV.

The bacterial diversity increased from cluster KRST-I (median Shannon index of 1.0) to cluster KRST-II (1.2) to clusters KRST-III, IV and V (2.1, 2.0, and 2.0, respectively; p <0.01). The evenness based on the five most abundant genera in each cluster was lower in clusters KRST-I and KRST-II compared to cluster KRST-pIII-V (Figure 1a). The richness in clusters KRST-I and KRST-II (a median of four and five genera per sample) was lower than in clusters KRST-III, IV and V (16, 14, and 14 genera per sample, respectively) (Figure 1b).

### Sociodemographic, behavioral and reproductive history correlates of VMB clusters

VMB clustering was not associated with sociodemographic, behavioral or reproductive history characteristics, with the exception of time elapsed since last delivery (Table 1). With every additional month since last delivery, women were less likely to be assigned to cluster KRST-II than cluster KRST-pIII-V (p = 0.01). We did not conduct multivariable modeling with this group of variables because few of them were significant at p ≤ 0.01 in bivariable models.

**Table 1:** **Sociodemographic, behavioral, and reproductive history correlates of VMB clusters**

**Correlates**		**KRST-I n (%) N = 19**	**KRST-II n (%) N = 136**	**KRST-pIII-V n (%) N = 53**	**p value** ^*^
*Sociodemographic characteristics at screening*					
Recruitment group:					
	Adult women, KE	9 (47.4)	60 (44.1)	21 (39.6)	0.69
	Adolescent women, KE	1 (5.3)	14 (10.3)	4 (7.5)	
	Pregnant women, KE	3 (15.8)	9 (6.6)	2 (3.8)	
	Adult women, RSA	2 (10.5)	22 (16.2)	7 (13.2)	
	IVP users, RSA + TZ	2 (10.5)	10 (7.4)	8 (15.1)	
	HIV-positive and high risk women, RW	2 (10.5)	21 (15.4)	11 (20.8)	
Country:					
	Kenya	13 (68.4)	83 (61.0)	27 (50.9)	0.69
	RSA	2 (10.5)	22 (16.2)	8 (15.1)	
	Rwanda	2 (10.5)	21 (15.4)	11 (20.8)	
	Tanzania	2 (10.5)	10 (7.4)	7 (13.2)	
Age (categorical):					
	16 -17 years	1 (5.3)	14 (10.3)	4 (7.5)	0.18
	18 -24 years	3 (15.8)	50 (36.8)	20 (37.7)	
	25 -29 years	6 (31.6)	48 (35.3)	17 (32.1)	
	30 -35 years	9 (47.4)	24 (17.6)	12 (22.6)	
Socio-economic status (composite)^a^:					
	Low	6 (31.6)	42 (30.9)	15 (28.3)	0.58
	Medium	5 (26.3)	44 (32.4)	23 (43.4)	
	High	8 (42.1)	50 (36.8)	15 (28.3)	
*Sexual behavior*					
Sexual risk taking (composite)^b^:					
	Low	7 (36.8)	55 (40.4)	22 (41.5)	0.63
	Medium	6 (31.6)	42 (30.9)	11 (20.8)	
	High	6 (31.6)	39 (28.7)	20 (37.7)	
Self-acknowledged sex-worker:					
	No	16 (84.2)	115 (84.6)	43 (81.1)	0.88
	Yes	3 (15.8)	21 (15.4)	10 (18.9)	
Number of lifetime sexual partners^c^:					
	One	7 (38.9)	29 (21.6)	10 (20.8)	0.55
	Two to three	7 (38.9)	55 (41)	20 (41.7)	
	More than three	4 (22.2)	50 (37.3)	18 (37.5)	
Number of sexual partners last 3 months^d^:					
	Zero	3 (15.8)	11 (8.2)	1 (1.9)	0.24
	One	12 (63.2)	101 (75.4)	42 (79.2)	
	> One	4 (21.1)	22 (16.4)	10 (18.9)	
Vaginal sex previous morning/evening^e^:					
	No	18 (94.7)	97 (71.9)	40 (75.5)	0.09
	Yes	1 (5.3)	38 (28.1)	13 (24.5)	
Frequency of sex last 3 months^f^:					
	≤10 times	7 (50.0)	45 (38.8)	20 (42.6)	0.21
	11 - 30 times	6 (42.9)	36 (31.0)	19 (40.4)	
	>30 times	1 (7.1)	35 (30.2)	8 (17.0)	
Condom use last sexual contact^f^:					
	No	13 (81.2)	70 (60.3)	32 (68.1)	0.24
	Yes	3 (18.8)	46 (39.7)	15 (31.9)	
Freq of unprotected sex (last 3 months)^g^:					
	Never	0 (0.0)	36 (60.0)	12 (63.2)	0.06
	<10 times	2 (40.0)	13 (21.7)	4 (21.1)	
	≥10 times	3 (60.0)	11 (18.3)	3 (15.8)	
Put something in vagina to dry/tighten vagina before sex (enrollment)^h^					
	No	17 (100.0)	125 (99.2)	46 (100.0)	1.00
	Yes	0 (0.0)	1 (0.8)	0 (0.0)	1.00
Clean vagina after sex (enrollment):					
	No	7 (36.8)	67 (49.3)	28 (52.8)	0.50
	Yes	12 (63.2)	69 (50.7)	25 (47.2)	
Vaginal practice for washing:					
	No	7 (36.8)	51 (37.5)	21 (39.6)	0.88
	Yes, but not the evening before visit	3 (15.8)	14 (10.3)	7 (13.2)	
	Yes, including evening before visit	9 (47.4)	71 (52.2)	25 (47.2)	
Products used to wash/clean/dry vagina					
	No product	6 (31.6)	51 (37.5)	20 (37.7)	0.48
	Water or fingers only	6 (31.6)	39 (28.7)	17 (32.1)	
	Water + soap	3 (15.8)	35 (25.7)	14 (26.4)	
	Cloth	4 (21.1)	11 (8.1)	2 (3.8)	
*Reproductive history at screening*					
Median gravidity (IQR)		2 (1, 3)	2 (1, 3)	2 (1, 3)	0.78
Median parity (IQR)		2 (1, 2.5)	1.5 (0, 2)	1 (0, 2)	0.76
Currently pregnant					
	No	16 (84.2)	127 (93.4)	51 (96.2)	0.19
	Yes	3 (15.8)	9 (6.6)	2 (3.8)	
Breast feeding:					
	No	16 (84.2)	119 (87.5)	49 (92.5)	0.48
	Yes	3 (15.8)	17 (12.5)	4 (7.5)	
Median months since last delivery^i†^ (IQR)		41 (28, 54)	33 (19, 48)	56 (28, 82)	0.01
Current contraceptive use:					
	None, pregnant	3 (15.8)	9 (6.6)	2 (3.8)	0.58
	None, not pregnant	3 (15.8)	30 (22.1)	13 (24.5)	
	Combined oral contraceptives	3 (15.8)	19 (14.0)	9 (17.0)	
	Progestin-only injectables	5 (26.3)	44 (32.4)	11 (20.8)	
	Condoms + IUD	5 (26.3)	34 (25.0)	18 (34.0)	

*Abbreviations:* *IQR* inter quartile range, *IUD* intra uterine device, *IVP* intravaginal practice, *KE* Kenya, *KRST* Kenya, Rwanda, South Africa and Tanzania, *RSA* Republic of South Africa, *RW* Rwanda, *TZ* Tanzania.

*Kruskal-Wallis test for continuous data and Fisher’s exact test for categorical data.

^a^The composite score ‘socio-economic-status’ was calculated as follows: income: no income (=1), up to the median (=2), median to 75^th^ percentile (=3), and ≥ 75^th^ percentile (=4); housing: informal dwelling (=1), room inside house or flat (=2), rented house or flat (=3), bonded/mortgaged house or flat (=4); and toilet: no facility/bush/field/yraditional pit toilet (=1), ventilated improved pit latrine (=2), and flush toilet (=3). The total score was categorized in tertiles as low, medium, high.

^b^The composite variable for sexual risk taking was constructed as follows: High risk: sex worker OR at least three sex partners last year OR had at least one sex partner (in the last 3 months) with HIV OR age first sex less than 15 yrs; Medium risk: at least two sex partners last year OR had at least one sex partner (in the last 3 months) who had multiple partners; Low risk: one or no sex partners in last year AND did not have any sex partner (in the last 3 months) with multiple partners AND age first sex at least 15 years.

^c^8/208 values missing.

^d^2/208 values missing.

^e^1/208 values missing.

^f^30/208 values missing.

^g^124/208 values missing.

^h^19/208 values missing.

^i^71/208 values missing.

^†^For every one month increase in the time since last delivery, women were less likely to be assigned to cluster KRST-II than cluster KRST-pIII-V (OR = 0.98; 95% CI 0.97, 0.99). Time since last delivery was not statistically significantly different between clusters KRST-I and KRST-II, and between clusters KRST-I and KRST-pIII-V.

### Associations between VMB clusters, Nugent scores and Amsel criteria

**Figure 3:**
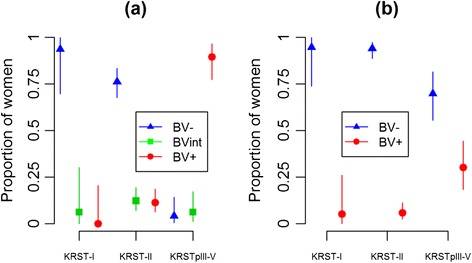
**BV status distribution by Nugent score (a) and Amsel criteria (b) in the three VMB clusters.** In Figure 3a, women were classified as BV-negative (BV-), BV intermediate (BVint), or BV-positive (BV+) based on Nugent score of 0–3, 4–6 and 7–10, respectively. In Figure 3b, women were classified as BV-positive if any three of the four Amsel criteria (i.e. clue cells > 20%, positive whiff test, vaginal pH > 4.5, and/or presence of unusual vaginal discharge) was positive. The error bars correspond to the 95% confidence interval of the proportions.

Diagnosis of BV by Nugent score and Amsel criteria were each strongly associated with VMB clustering (p < 0.0001) (Table 2, Figure 3). The majority (>88%) of women in clusters KRST-I and II were diagnosed as BV-negative by Nugent score and Amsel criteria. However, while the majority of women (89.6%) in cluster KRST-pIII-V was diagnosed as BV-positive by Nugent score, only 30% were diagnosed as BV-positive by Amsel criteria. Only 9.6% of women were diagnosed with intermediate microbiota by Nugent score and approximately equal proportions were found in each VMB cluster (Figure 3).

**Table 2:** **Clinical correlates of VMB clusters.**

**Correlates**		**KRST-I n (%) N = 19**	**KRST-II n (%) N = 136**	**KRST-pIII-V n (%) N = 53**	**p value** *
*Self reported symptoms at screening*					
Vaginal discharge reported:					
No		18 (8.9)	135 (66.5)	50 (24.6)	0.05
Yes		1 (20.0)	1 (20.0)	3 (60.0)	
*Clinician observed findings at screening*					
Cervical mucus^ab†^:					
No		5 (4.2)	82 (69.5)	31 (26.3)	0.04
Mild		11 (14.1)	48 (61.5)	19 (24.4)	
Abundant		2 (28.6)	3 (42.9)	2 (28.6)	
Cervical and/or vaginal epithelial abnormalities:					
No		15 (8.0)	124 (66.3)	48 (25.7)	0.26
Yes		4 (19.0)	12 (57.1)	5 (23.8)	
Ectopy:					
No		11 (9.4)	73 (62.4)	33 (28.2)	0.57
Yes		8 (8.8)	63 (69.2)	20 (22)	
Any colposcopy finding?:					
No		17 (9.4)	119 (65.7)	45 (24.9)	0.90
Yes		2 (7.4)	17 (63.0)	8 (29.6)	
*Laboratory-confirmed reproductive and urinary tract infections*					
HIV serology					
Negative		19 (100.0)	128 (94.8)	50 (94.3)	0.78
Positive		0 (0.0)	7 (5.2)	3 (5.7)	Trend: 0.44
HSV-2 serology					
Negative		15 (78.9)	85 (62.5)	33 (62.3)	0.36
Positive		4 (21.1)	51 (37.5)	20 (37.7)	Trend: 034
Bacterial STI^c^					
Negative		17 (94.4)	116 (83.3)	41 (77.4)	0.20
Positive		1 (5.6)	20 (14.7)	12 (22.6)	Trend: 0.07
BV by Nugent score					
0-3		15 (93.8)	93 (76.2)	2 (4.2)	<0.01
4-6		1 (6.2)	15 (12.3)	3 (6.3)	
7-10		0 (0.0)	14 (11.5)	43 (89.6)	
BV by Amsel criteria					
Negative		18 (94.7)	128 (94.1)	37 (69.8)	<0.01
Positive		1 (5.3)	8 (5.9)	16 (30.2)	
Candidiasis on wet mount^d^					
Negative		16 (84.2)	113 (83.1)	50 (94.3)	0.11
Positive		3 (15.8)	23 (16.9)	3 (5.7)	Trend: 0.09
Urinary tract infection by diptstick test					
Negative		4 (100.0)	4 (44.4)	132 (76.3)	0.06
Positive		0 (0.0)	5 (55.6)	41 (23.7)	
*Treatment in the 14 days prior to enrollment*					
Any systemic antibiotics^d^:					
No		18 (10.5)	115 (66.9)	39 (22.7)	0.10
Yes		1 (2.8)	21 (58.3)	14 (38.9)	
Any vaginal antibiotics: No					
No		19 (9.2)	136 (66)	51 (24.8)	0.12
Yes		0 (0)	0 (0)	2 (100)	
Bacterial vaginosis requiring treatment:					
No		19 (9.7)	130 (66.3)	47 (24.0)	0.16
Yes		0 (0)	6 (50.0)	6 (50.0)	
Candidiasis requiring treatment^e^:					
No		17 (9.5)	116 (64.8)	46 (25.7)	0.10
Yes		0 (0)	10 (100)	0 (0)	

*Abbreviations:* *KRST* Kenya, Rwanda, South Africa, and Tanzania.

*Fisher’s exact test, Trend = Chi-squared test for trend.

^a^4/208 values missing.

^b^No association (p = 0.95) was found when the data were analyzed with a binary outcome (i.e. clusters KRST-I and KRST-II combined vs. cluster KRST-pIII-V).

^c^Bacterial STIs includes syphilis (by serology), chlamydia and gonorrhea (by PCR), and trichomoniasis (by InPouch culture test).

^d^Stronger evidence of association was observed when data were analyzed with a binary outcome (i.e. clusters KRST-I and II combined vs. cluster KRST-pIII-V) (p = 0.06) and when pregnant women and Tanzanian women (phase in the menstrual cycle at the time of sampling not known) were excluded (p = 0.04).

^e^19/208 values missing.

^†^Women who had mild/moderate cervical mucus (OR = 0.27; 95% CI 0.09, 0.81) and women who had abundant cervical mucus (OR = 0.09; 95% CI 0.01, 0.68) were more likely to belong to cluster KRST-II vs. KRST-I compared to women with no cervical mucus. Women who had mild cervical mucus compared to no cervical mucus were less likely to belong to cluster KRST-pIII-V vs. KRST-I (OR = 0.28; 95% CI 0.08, 0.93), while there was no statistically significant difference between having abundant cervical mucus and no cervical mucus.

### Clinical correlates of VMB clusters

The proportions of women with laboratory-confirmed bacterial STIs (5.6% in KRST-I, 14.7% in KRST-II and 22.6% in KRST-pIII-V), HSV-2 (21.1%, 37.5%, and 37.7%) and HIV (0%, 5.2%, and 5.7%) increased from cluster KRST-I to KRST-pIII-V, indicating a trend although the evidence for an association was weak (p_trend_ = 0.07 for STIs, 0.34 for HSV2 and 0.44 for HIV) (Table 2, Additional file 1: Figure S2). No women in cluster KRST-I had an UTI compared to 55.6% in cluster KRST-II and 23.7% in cluster KRST-pIII-V (p = 0.06; Table 2). Women with abundant cervical mucus upon speculum examination were more likely to belong to cluster KRST-I, but the total number of women with abundant cervical mucus was small (p = 0.04; Table 2). Women who reported unusual vaginal discharge were more likely to belong to cluster KRST-pIII-V (p = 0.05; Table 2). There was no evidence for an association between VMB clustering and any other clinical characteristics. We did not conduct multivariable modeling with this group of variables due to the fact that few of them were significant at p ≤ 0.01 in bivariable models.

### Immunological correlates of VMB clusters

The bivariable models showed strong evidence for an increase of the pro-inflammatory cytokines and growth factors IL-1α, IL-1β, G-CSF, and GM-CSF, and a decrease of IP-10, when moving from cluster KRST-I to KRST-pIII-V (Table 3). The MIP-1β concentration was highest in cluster KRST-II and lowest in cluster KRST-I. Six variables (IL-1α, IL-1β, G-CSF, GM-CSF, IP-10, and MIP-1β) qualified for inclusion in the multivariable model, but the final model (AIC = 296) included only four of them (IL-1α, G-CSF, IP-10, and MIP-1β). IL-1β, which was highly significant in the bivariable analysis, was eliminated due to its high correlation with IL-1α (Pearson’s correlation coefficient = 0.65). The associations in the final model were in the same direction as in the bivariable models.

**Table 3:** **Immunological correlates of VMB clusters**

**Correlates**	**KRST-I (n = 19)**		**KRST-II (n = 136)**			**KRST-pIII-V (n = 53)**			**p value** ^*a^
**(in log10)**	**Mean (95% CI)**	**OR/aOR (95% CI)**	**Mean (95% CI)**	**OR (95% CI)**	**aOR (95% CI)**	**Mean (95% CI)**	**OR (95% CI)**	**aOR (95% CI)**	
IL-1α	1.08 (0.86, 1.3)	Ref	1.22 (1.14, 1.31)	1.61 (0.69, 3.77)	1.58 (0.58, 4.33)	1.64 (1.48, 1.81)	7.4 (2.63, 20.82)	11.5^†^ (3.29, 40.22)	<0.0001
IL-1β	0.65 (0.33, 0.97)	Ref	0.83 (0.71, 0.95)	1.4 (0.72, 2.74)	-	1.34 (1.11, 1.57)	3.33 (1.58, 7.03)	-	<0.0001
IL-1ra^b^	4.65 (4.36, 4.94)	Ref	4.83 (4.74, 4.91)	1.9 (0.76, 4.76)	-	4.87 (4.74, 5)	2.28 (0.79, 6.6)	-	0.28
IL-6	0.64 (0.33, 0.95)	Ref	0.9 (0.79, 1)	1.68 (0.85, 3.3)	-	0.9 (0.68, 1.11)	1.68 (0.8, 3.54)	-	0.31
IL-8	2.06 (1.83, 2.28)	Ref	2.19 (2.09, 2.28)	1.41 (0.62, 3.2)	-	2.25 (2.04, 2.45)	1.65 (0.68, 3.97)	-	0.54
IL-12^c^	0.07 (−0.18, 0.32)	Ref	0.13 (0.05, 0.21)	1.31 (0.45, 3.81)	-	0.24 (0.12, 0.35)	2.11 (0.66, 6.7)	-	0.28
IP-10	2.54 (2.36, 2.73)	Ref	2.62 (2.51, 2.73)	1.22 (0.57, 2.6)	0.98 (0.39, 2.43)	2.15 (1.93, 2.38)	0.47 (0.21, 1.06)	0.16^†^ (0.05, 0.47)	0.0002
GM-CSF	0.26 (0.05, 0.47)	Ref	0.32 (0.25, 0.39)	1.38 (0.47, 4.03)	-	0.13 (−0.01, 0.27)	0.58 (0.19, 1.78)	-	0.0351
G-CSF	1.84 (1.57, 2.11)	Ref	1.91 (1.79, 2.02)	1.16 (0.57, 2.38)	0.71 (0.26, 1.92)	2.08 (1.93, 2.23)	1.83 (0.81, 4.15)	3.6^††^ (1.02, 12.73)	0.18
MIP-1β^d^	0.69 (0.23, 1.15)	Ref	0.94 (0.83, 1.05)	1.57 (0.84, 2.94)	1.73 (0.79, 3.82)	0.7 (0.48, 0.93)	1.02 (0.52, 1.98)	0.61 (0.25, 1.52)	0.085

*Abbreviations:* *CI* confidence interval, *KRST* Kenya, Rwanda, South Africa, Tanzania, *OR* odds ratio, *aOR* adjusted odds ratio. For cytokine and growth factor abbreviations, we refer to the text.

*One way analysis of variance test in a bivariable analysis.

^a^Six variables qualified for inclusion in the multivariable model but only four (IL-1α, IP-10, G-CSF, and MIP-1β) variables remained in the final model after the model selection process. Although, IL-1β was highly significant in the bivariable analysis, it was eliminated during the model selection because it was highly correlated with IL-1α (Pearson’s correlation coefficient = 0.65).

^b^19/208 values missing.

^c^Stronger evidence of association was observed when pregnant women and Tanzanian women (stage of menstrual cycle at time of sampling not known) were excluded (p = 0.09).

^d^Weaker evidence of association was observed when pregnant women and Tanzanian women (stage of menstrual cycle at time of sampling not known) were excluded (p = 0.27).

^†^Adjusted P-value < 0.001.

^††^Adjusted P-value = 0.01.

## Discussion

We identified five vaginal microbiota clusters in a diverse group of women from four African countries. Two of these clusters were dominated by *L. crispatus* or *L. iners*, while the other three clusters consisted of various combinations of other (facultative) anaerobic bacteria in addition to *L. iners.* Studies in the USA, Europe and Asia have also reported clusters dominated by *L. crispatus* and *L. iners*, but *L. crispatus* clusters were typically more common and *L. iners* clusters less common than in our study [8,10,28,29]. Longitudinal studies have shown that *L. crispatus* protects women from vaginal dysbiosis more efficiently than *L. iners* (reviewed in 6). We did not identify any clusters dominated by *L. gasseri* or *L. jensenii*. Such clusters have been reported in studies in the USA, Europe and Asia but were less common than clusters dominated by *L. crispatus* or *L. iners* [8,10,28,29]. We found that Nugent scoring correlated well with molecular VMB clustering but the Amsel criteria did not, as has been found by others [reviewed in 6]. This may have clinical relevance since the Amsel criteria are often used in clinic settings to diagnose BV.

Women in the pooled dysbiotic cluster KRST-pIII-V had higher concentrations of several pro-inflammatory factors than women in clusters KRST-I and II. In the past, BV has often been described as a non-inflammatory syndrome, but more recent data consistently show that vaginal dysbiosis is associated with subclinical cervicovaginal immune activation [19,30]. The differences in inflammatory markers could also partially be explained by hormonal differences between the VMB clusters such as stage of the menstrual cycle, use of hormonal contraception and pregnancy. A recent systematic review showed that hormonal contraceptive use and pregnancy are negatively associated with vaginal dysbiosis [31], and it has been suggested that pregnancy is associated with a pro-inflammatory state [32]. However, in this study, hormonal contraceptive use and pregnancy were not associated with VMB clustering and a sensitivity analysis excluding the pregnant women and women from Tanzania (for whom we did not know the stage of the menstrual cycle at the time of sampling) did not change our results substantively (see footnotes Table 3). Inflammation related to vaginal dysbiosis is of concern because vaginal dysbiosis is very common (25.5% in this study) [33] and inflammation in the genital tract results in the attraction of CD4+ target cells for HIV [20] as well as shedding of HIV in HIV-positive women [34]. There was no evidence for an association between VMB clustering and HIV prevalence in this study (likely due to the small number of HIV infections) but we recently showed a strong association (using the same phylogenic microarray as in this study) in female sex workers in Kigali, Rwanda [3].

There was some evidence that a lower proportion of women in cluster KRST-I tested positive for STIs (p_trend_ = 0.07) and UTIs (p = 0.06) than in the other clusters, but the number of cases was small. These findings are, however, consistent with the findings of the above-mentioned study in female sex workers in Kigali that reported negative associations between a *L. crispatus*-dominated VMB and various STIs [3]. Other studies have also shown that lactobacilli-dominated VMB [reviewed in 6] or a Nugent score 0–3 [4,5,35] are negatively associated with both viral and bacterial STIs. The relationship between the VMB and UTIs has not been adequately studied, but recent studies have shown that the urine microbiome in women resembles their VMB [36]. In contrast, a higher proportion of women in clusters KRST-I and II had vaginal candidiasis than women in the other clusters (p_trend_ = 0.09), and this is consistent with findings of several other studies (reviewed in [6]).

We identified few additional correlates of VMB composition, perhaps due to limited statistical power. Women who had delivered a baby more recently were more likely to belong to clusters KRST-I and KRST-II (p = 0.01), whereas women who reported unusual vaginal discharge were more likely to belong to clusters KRST-III/IV/V (p = 0.05). The former could be explained by sex hormone levels but this seems unlikely since the median time period since the last delivery was at least 33 months in all three groups. Confounding by age or sexual behavior might be more likely. There was some evidence that women in the KRST-I cluster were older, had fewer sexual partners in the last three months, but more unprotected sex with a steady partner, than women in the other clusters.

Our study used samples and data from two studies. While we used the same VMB assessment methods in both studies, there were some differences in other assessments such as the way questions were asked, the test kits that were used for on-site diagnostic testing, and the platforms that were used in Antwerp and London for cytokine testing [37]. Other limitations include the cross-sectional nature of the study, the small sample sizes in some of the comparison groups (particularly the number of HIV and bacterial STI cases), and the imprecise timing of sexual behaviors (in the Vaginal Biomarkers Study), STI and UTI diagnoses (in the Vaginal Biomarkers Study) and stage of the menstrual cycle (in the Tanzania study) relative to microarray sampling.

## Conclusions

Vaginal dysbiosis in African women was significantly associated with vaginal inflammation. The associations with increased prevalence of STIs (including HIV) and UTIs, and decreased prevalence of vaginal candidiasis, should be confirmed in larger studies.

## Additional file

Additional file 1:
**Supplementary methods, tables and figures.**
