# Supplementary material for: Correlates of the molecular vaginal microbiota composition of African women
Source: BMC Infect Dis. 2015 Feb 21;15:86. doi: 10.1186/s12879-015-0831-1 (PMC4343073; doi:10.1186/s12879-015-0831-1)
Supplement: Additional file 1: — Supplementary methods, tables and figures. [file 12879_2015_831_MOESM1_ESM.docx]

**ADDITIONAL FILE 1**

**Additional methods, tables and figures**

**Manuscript Title: Correlates of the molecular vaginal microbiota composition of African women**

**Authors:** Raju Gautam, Hanneke Borgdorff, Vicky Jespers, Suzanna C. Francis, Rita Verhelst, Mary Mwaura, Sinead Delany-Moretlwe, Gilles Ndayisaba, Jordan K. Kyongo, Liselotte Hardy, Joris Menten, Tania Crucitti, Evgeni Tsivtsivadze, Frank Schuren, Janneke H.H.M. van de Wijgert, for the Vaginal Biomarkers Study Group.

**Methods**

The three study sites of the Vaginal Biomarkers Study were: 1) The International Centre for Reproductive Health Kenya (ICRHK) in Mombasa, Kenya; 2) Rinda Ubuzima in Kigali, Rwanda; and 3) The Wits Reproductive Health and HIV Research Institute (WRHI) in Johannesburg, Republic of South Africa (RSA). The Tanzania Study was conducted at the Mwanza Intervention Trials Unit at the Tanzanian National Institute of Medical Research, Mwanza, Tanzania.

The studies were approved by the following ethics committees: the Kenyatta National Hospital Ethical Review Committee, Kenya; the Human Research Ethics Committee (Medical), University of the Witwatersrand, RSA; the Rwanda National Ethics Committee, Rwanda; the Medical Research Coordinating Committee of the Tanzanian National Institute for Medical Research; and the Institutional Review Boards of the Institute of Tropical Medicine in Antwerp, the University Teaching Hospital in Antwerp, and Ghent University, Belgium, and the London School of Hygiene and Tropical Medicine, UK. In addition the study was approved by the National Council on Science and Technology in Kenya; the RSA Department of Health; and the National AIDS Control Commission in Rwanda. Study participants received a modest reimbursement for each scheduled study visit as follows: the equivalent in local currency of 7 USD in Kenya, 9 USD in Rwanda, 14 USD in South-Africa, and 3 USD in Tanzania.

**Supplementary Table S1: Details of diagnostic laboratory tests used at each study site**

|  | **RU, Kigali, Rwanda** | **ICRHK, Mombasa, Kenya** | **WrHI, Johannesburg, RSA** | **MITU/NIMR, Mwanza, Tanzania** |
| --- | --- | --- | --- | --- |
| **HIV** | 1. Determine 2. SD Bioline 3. UniGold Recombigen | 1. Determine 2. SD Bioline 3. UniGold Recombigen | 1. Determine 2. UniGold Recombigen 3. ELISA 4^th^ generation | 1. Determine 2. SD Bioline 3. Murex HIV 1.2.O and Vironostika HIV Uniform II plus O 4. P24 Antigen 5. Western Blot |
| **Syphilis** | Spinreact/Human RPR test followed by SerodiaTPPA to confirm reactive result. | BD Macrovue RPR test followed by SerodiaTPPA to confirm reactive result. | BD Macrovue RPR test followed by Serodia TPPA to confirm reactive result. | Omega RPR card test followed by SerodiaTPPA. |
| **HSV-2** | Kalon Biological HSV2 IgG ELISA | | | |
| **CT/NG** | Abbott RealTime CT/NG PCR | Dia-CT/NG real time PCR | BD ProbeTec ET CT/NG Amplified DNA assay | COBAS Amplicor CT/NG PCR. All positive tests for NG were confirmed by in-house PCR at ITM |
| ***Trichomonas vaginalis*** | Wet Mount Microscopy, InPouch TV | | | InPouch TV. Positives confirmed by in-house PCR at ITM. |
| **Microscopy** | Wet Mount Microscopy + Whiff test  Nugent Scoring: 2 slides – 1 done at site, 1 done at ITM (ITM result used for analysis) | | | |
|  |  |  |  |  |
| **Vaginal pH** | Machery-Nagel pH Fix 3.6-6.1 | | | Thermofisher pH test strip (3.6-6.1) |
| **Urine dipstick** | Siemens Multistix 10SG Reagent Strips | Mission Urinalysis Reagent strips | Neotest 4 urine dipstick | Not done |
| **Urine pregnancy** | QuickVue One-Step hCG test | Unimed First Sign hCG test | QuickVue One-Step hCG test | Local pregnancy test validated against QuickVue One-Step hCG test |

Laboratorium assay manufacturers in order of appearance in Table S1:

| Determine HIV-1/2: Abbott Diagnostic Division, Hoofddorp, The Netherlands.  SD Bioline HIV-1/2: Standard Diagnostics Inc., Kyonggi-do, South Korea.  Uni-Gold Recombigen HIV-1/2: Trinity, Berkeley Heights, New Jersey, USA.  ELISA 4^th^ generation: Abbott Diagnostic Division, Hoofddorp, The Netherlands.  Murex HIV 1.2.O: Abbott Murex, Wiesbaden, Germany.  Vironostika HIV Uniform II plus O: bioMérieux, Basingstoke, Hampshire, UK.  Spinreact/Human RPR: Spinreact Reactivos, Girona, Spain.  BD Macro-Vue RPR: Becton, Dickinson and Co, Maryland, USA.  Omega RPR card test: Omega Diagnostics, Alva, Scotland.  Serodia TPPA: Fujirebio Diagnostics, Pennsylvania, USA and Tokyo, Japan.  Kalon HSV-2 IgG ELISA: Kalon Biologicals, Aldershot, UK. | Abbott RealTime CT/NG: Abbott Diagnostic Division, Hoofddorp, The Netherlands.  Dia-CT/NG real-time PCR: Diagenode Diagnostics, Liège, Belgium.  BD ProbeTec ET CT/NG Amplified DNA Assay: BD Diagnostic Systems, Sparks, MD, USA.  COBAS Amplicor CT/NG PCR: Roche Diagnostics, Branchburg, NJ, USA.  InPouch TV: Biomed Diagnostics, White City, OR, USA.  Machery-Nagel pH Fix 3.6-6.1: Machery-Nagel, Düren, Germany.  ThermoFisher pH test strip 3.6-6.1: ThermoFisher, Waltham, MA, USA.  Siemens Multistix 10SG Reagent Strips: Siemens Healthcare, The Hague, Netherlands  Mission Urinalysis Reagent strips: ACON Laboratories, San Diego, CA, USA.  Neotest 4 urine dipstick: Kendon Medical Supplies, Johannesburg, RSA  QuickVue One-Step hCG pregnancy test: Quidel Corporation, San Diego, CA, USA.  Unimed FirstSign hCG pregnancy test: Unimed, International, Santa Clara, CA, USA. |
| --- | --- |

**Results**

*Supplementary Figure S1* shows the results of the clustering analysis using data from 313 vaginal samples tested on the microarray. We identified five VMB clusters, which are visualized in a co-occurrence matrix (Supplementary Figures S1A-C). The probabilistic decomposition of this matrix (Supplementary Figure S1C), which returns the probability of each sample belonging to a specific cluster, confirms the presence of these five clusters. Samples with a probability of <70% of belonging to one of these five clusters are shown in white in the top bar (Supplementary Figure S1A). Thirty-five samples from 22 women had a probability of <70% belonging to one of the five clusters. These samples did not cluster together, and the sociodemographic, behavioral and clinical characteristics of the 22 women did not differ significantly from those of the 208 women who were assigned to a cluster (*Supplementary Tables S2 and S3*).

*Supplementary Figure S2* shows the distribution of the proportion of women with laboratory-confirmed bacterial or viral STIs, UTI, or vaginal candidiasis across the three cervicovaginal microbiome clusters. These data are also presented in the manuscript but are presented here visually.


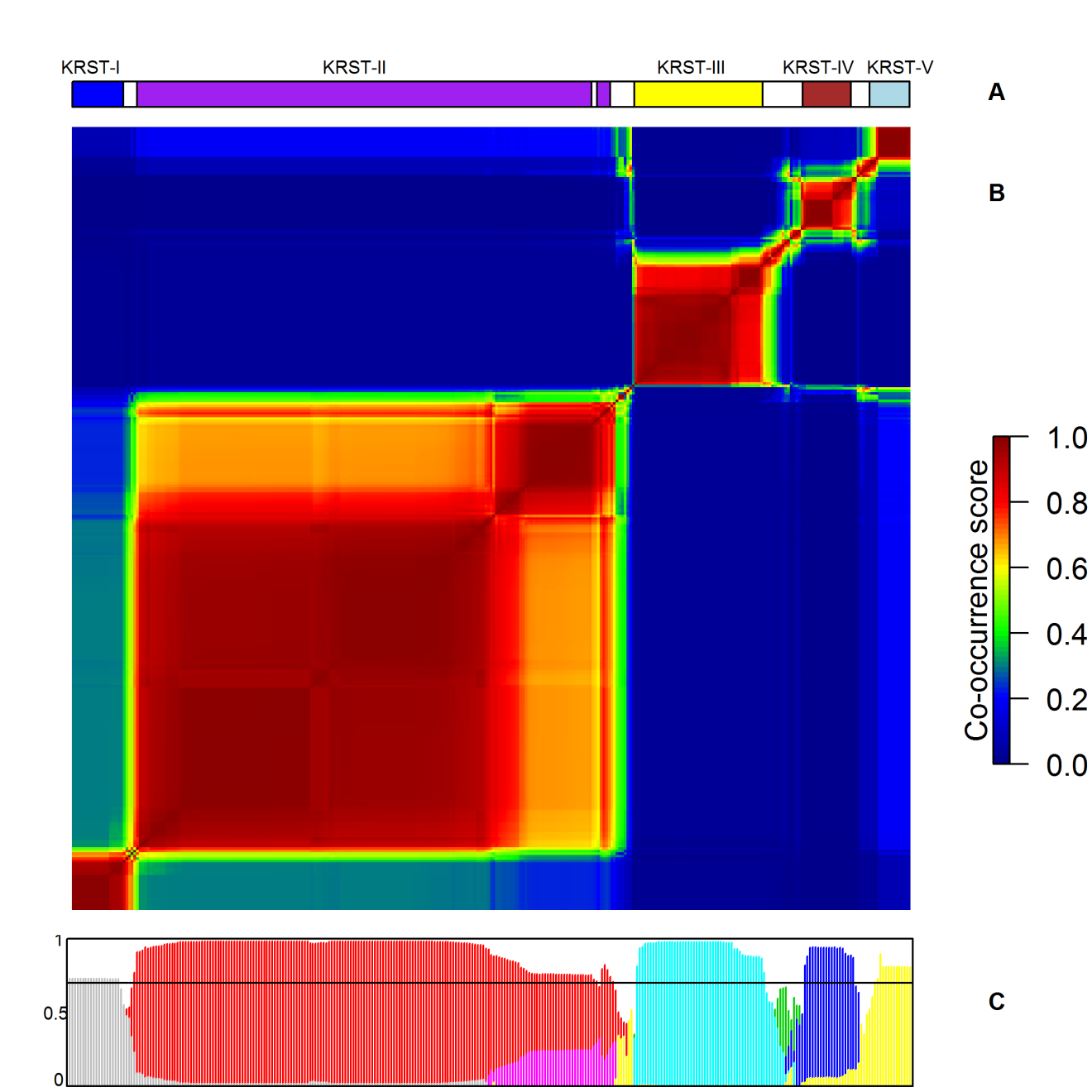


**Supplementary Figure S1**. Clustering of 313 vaginal samples tested on the microarray. The clusters were obtained by Neighborhood Co-regularized Multi-view Spectral Clustering. Only samples with at least 70% probability of belonging to a cluster are assigned **(A, C)**; unassigned samples appear in white in the top bar **(A)**. The matrix **(B)** shows the co-occurrence score (i.e. how many times samples co-occurred in different clustering runs) of the 313 samples on both the x-and y-axis in the same order.

**Supplementary Figure S2.** Distribution of the proportion of women with laboratory-confirmed bacterial or viral STIs, UTI, or vaginal candidiasis across the three cervicovaginal microbiome clusters. The proportions of women per cluster with the following infections were: laboratory-confirmed bacterial STIs (1/18=5.6% in KRST-I, 20/136=14.7% in KRST-II and 12/53=22.6% in KRST-pIII-V; ^■^Chi-square trend p-value=0.07), Candida (3/19=15.8%, 23/136=16.9% and 3/53=5.7%; ^†^Chi-square trend p-value=0.09), HIV (0/19=0%, 7/135=5.2% , and 3/53=5.7%; Chi-square trend p-value=0.44), HSV-2 (4/19=21.1% , 51/136=37.5% , and 20/53=37.7%; Chi-square trend p-value=0.34), and UTIs (0/4=0%, 5/9=55.6% and 41/173=23.7%; ^+^Fisher’s exact p-value=0.06). *There were no HIV or UTI-positive women in cluster KRST-I.

**Supplementary Table S2: Sociodemographic, behavioural, and reproductive history correlates of VMB clusters**

| Description | | KRST-I  n (%) (N = 19) | KRST-II  n (%) (N = 136) | KRST-p-III-V  n (%)  (N = 53) | KRST-UA  n (%)  (n = 22) | P value^*^ |
| --- | --- | --- | --- | --- | --- | --- |
| *Sociodemographic characteristics at screening* | | | | | | |
| Recruitment group: Adult women Kenya | | 9 (9) | 60 (60) | 21 (21) | 10 (10) | 0.91 |
|  | Adolescents Kenya | 1 (4.8) | 14 (66.7) | 4 (19) | 2 (9.5) |  |
|  | Pregnant women Kenya | 3 (20) | 9 (60) | 2 (13.3) | 1 (6.7) |  |
|  | Adult women South Africa | 2 (6.1) | 22 (66.7) | 7 (21.2) | 2 (6.1) |  |
|  | Intravaginal practices South Africa + Tanzania | 2 (8.7) | 10 (43.5) | 8 (34.8) | 3 (13) |  |
|  | HIV-positive and high risk women Rwanda | 2 (5.3) | 21 (55.3) | 11 (28.9) | 4 (10.5) |  |
| Country: Kenya | | 13 (9.6) | 83 (61) | 27 (19.9) | 13 (9.6) | 0.88 |
|  | South Africa | 2 (5.6) | 22 (61.1) | 8 (22.2) | 4 (11.1) |  |
|  | Rwanda | 2 (5.3) | 21 (55.3) | 11 (28.9) | 4 (10.5) |  |
|  | Tanzania | 2 (10) | 10 (50) | 7 (35) | 1 (5) |  |
| Age (categorical) 16-17 years | | 1 (4.8) | 14 (66.7) | 4 (19) | 2 (9.5) | 0.11 |
|  | 18-24 years | 3 (3.9) | 50 (65.8) | 20 (26.3) | 3 (3.9) |  |
|  | 25-29 years | 6 (7.5) | 48 (60) | 17 (21.2) | 9 (11.2) |  |
|  | 30-35 years | 9 (17) | 24 (45.3) | 12 (22.6) | 8 (15.1) |  |
| Socio-economic status (composite)^a^: Low | | 6 (8.7) | 42 (60.9) | 15 (21.7) | 6 (8.7) | 0.79 |
|  | Medium | 5 (6.3) | 44 (55.7) | 23 (29.1) | 7 (8.9) |  |
|  | High | 8 (9.8) | 50 (61) | 15 (18.3) | 9 (11) |  |
| *Sexual behaviour* | | | | | | |
| Sexual risk taking (composite)^b^: Low | | 7 (7.7) | 55 (60.4) | 22 (24.2) | 7 (7.7) | 0.67 |
|  | Medium | 6 (8.8) | 42 (61.8) | 11 (16.2) | 9 (13.2) |  |
|  | High | 6 (8.5) | 39 (54.9) | 20 (28.2) | 6 (8.5) |  |
| Self-acknowledged sex worker: No | | 16 (8.3) | 115 (59.9) | 43 (22.4) | 18 (9.4) | 0.93 |
|  | Yes | 3 (7.9) | 21 (55.3) | 10 (26.3) | 4 (10.5) |  |
| Number of lifetime sexual partners: One | | 7 (14) | 29 (58) | 10 (20) | 4 (8) | 0.35 |
|  | Two to three | 7 (8) | 55 (63.2) | 20 (23) | 5 (5.7) |  |
|  | More than three | 4 (4.8) | 50 (59.5) | 18 (21.4) | 12 (14.3) |  |
| Number of sexual partners last 3 months: Zero | | 3 (20) | 11 (73.3) | 1 (6.7) | 0 (0) | 0.29 |
|  | Two to three | 12 (7) | 101 (58.7) | 42 (24.4) | 17 (9.9) |  |
|  | More than three | 4 (9.8) | 22 (53.7) | 10 (24.4) | 5 (12.2) |  |
| Vaginal sex previous morning or evening: No | | 18 (10.5) | 97 (56.7) | 40 (23.4) | 16 (9.4) | 0.17 |
|  | Yes | 1 (1.7) | 38 (65.5) | 13 (22.4) | 6 (10.3) |  |
| Frequency of sex last 3 months: ≤ 10 times | | 7 (8.4) | 45 (54.2) | 20 (24.1) | 11 (13.3) | 0.33 |
|  | 11 - 30 times | 6 (9) | 36 (53.7) | 19 (28.4) | 6 (9) |  |
|  | > 30 times | 1 (2.1) | 35 (72.9) | 8 (16.7) | 4 (8.3) |  |
| Condom use last sexual contact: No | | 3 (4.1) | 46 (62.2) | 15 (20.3) | 10 (13.5) | 0.25 |
|  | Yes | 13 (10.3) | 70 (55.6) | 32 (25.4) | 11 (8.7) |  |
| Frequency of unprotected sex last 3 months: Never | | 0 (0) | 36 (67.9) | 12 (22.6) | 5 (9.4) | 0.02 |
|  | <10 times | 2 (7.4) | 13 (48.1) | 4 (14.8) | 8 (29.6) |  |
|  | >= 10 times | 3 (16.7) | 11 (61.1) | 3 (16.7) | 1 (5.6) |  |

| Puts something in vagina to dry/tighten vagina before sex (enrollment) | | | |  |  |  |
| --- | --- | --- | --- | --- | --- | --- |
|  | No | 17 (8.2) | 125 (60.1) | 46 (22.1) | 20 (9.6) | 0.38 |
|  | Yes | 0 (0) | 1 (50) | 0 (0) | 1 (50) |  |
| Cleans vagina after sex: No | | 7 (6.2) | 67 (59.8) | 28 (25) | 10 (8.9) | 0.69 |
|  | Yes | 12 (10.2) | 69 (58.5) | 25 (21.2) | 12 (10.2) |  |
| Wash vagina: No | | 7 (8) | 51 (58) | 21 (23.9) | 9 (10.2) | 0.96 |
|  | Yes, but not the evening before visit | 3 (11.1) | 14 (51.9) | 7 (25.9) | 3 (11.1) |  |
|  | Yes, including evening before visit | 9 (7.8) | 71 (61.7) | 25 (21.7) | 10 (8.7) |  |
| Products used to wash/clean/dry vagina | |  |  |  |  |  |
|  | No product | 6 (7.1) | 51 (60) | 20 (23.5) | 8 (9.4) | 0.77 |
|  | Water or fingers only | 6 (8.7) | 39 (56.5) | 17 (24.6) | 7 (10.1) |  |
|  | Water + soap | 3 (5.3) | 35 (61.4) | 14 (24.6) | 5 (8.8) |  |
|  | Cloth | 4 (21.1) | 11 (57.9) | 2 (10.5) | 2 (10.5) |  |
| *Reproductive history at screening* | | | | | | |
| Median gravidity (IQR) | | 2 (1, 3) | 2 (1, 3) | 2 (1, 3) | 2 (1, 2.75) | 0.91 |
| Median parity (IQR) | | 2 (1, 2.5) | 1.5 (0, 2) | 1 (0, 2) | 1 (0.25, 2) | 0.85 |
| Currently pregnant: No | | 16 (7.4) | 127 (59.1) | 51 (23.7) | 21 (9.8) | 0.32 |
| Yes | | 3 (20.0) | 9 (60.0) | 2 (13.3) | 1 (6.7) |  |
| Currently breastfeeding: No | | 16 (7.9) | 119 (58.9) | 49 (24.3) | 18 (8.9) | 0.49 |
|  | Yes | 3 (10.7) | 17 (60.7) | 4 (14.3) | 4 (14.3) |  |
| Median months since last delivery (IQR) | | 41 (28, 54) | 33.15 (19, 48) | 55.75 (28, 82) | 42.1 (22, 78) | 0.04 |
| Current contraceptive use: None | |  |  |  |  |  |
|  | None, pregnant | 3 (20) | 9 (60) | 2 (13.3) | 1 (6.7) | 0.85 |
|  | None, not pregnant | 3 (6) | 30 (60) | 13 (26) | 4 (8) |  |
|  | Combined oral contraceptives | 3 (8.8) | 19 (55.9) | 9 (26.5) | 3 (8.8) |  |
|  | Progestin-only injectables | 5 (7.4) | 44 (64.7) | 11 (16.2) | 8 (11.8) |  |
|  | Condoms + IUD | 5 (7.9) | 34 (54) | 18 (28.6) | 6 (9.5) |  |

An expanded version of Table 1 in the manuscript, showing sociodemographic, behavioral and reproductive history characteristics of women in the three VMB clusters but also in the women not assigned to a cluster. "KRST" represents the first letter of the names of the four African countries where the study was conducted (Kenya, Rwanda, South Africa and Tanzania) and "KRST-pIII-V" denotes a new KRST cluster formed by collapsing KRST-III, KRST-IV and KRST-V. "UA" stands for unassigned. Other abbreviations: IQR=interquartile range, IUD=intrauterine device.

^*^Fisher’s exact test in bivariable analysis

^a^The composite score ‘socio-economic-status’ was calculated as follows: income: no income (=1), up to the median (=2), median to 75^th^ percentile (=3), and ≥ 75^th^ percentile (=4); housing: informal dwelling (=1), room inside house or flat (=2), rented house or flat (=3), bonded/mortgaged house or flat (=4); and toilet: no facility/bush/field/yraditional pit toilet (=1), ventilated improved pit latrine (=2), and flush toilet (=3). The total score was categorized as low, medium, high.

^b^The composite variable for sexual risk taking was constructed as follows: High risk: sex worker OR at least three sex partners last year OR had at least one sex partner (in the last 3 months) with HIV OR age first sex less than 15 yrs; Medium risk: at least two sex partners last year OR had at least one sex partner (in the last 3 months) who had multiple partners; Low risk: one or no sex partners in last year AND did not have any sex partner (in the last 3 months) with multiple partners AND age first sex at least 15 years.

**Supplementary Table S3: Clinical correlates of VMB clusters**

| Description | | KRST-I (n=19) | KRST-II (n=136) | KRST-p-III-V (n=53) | KRST-UA (n=22) | P value^*^ |
| --- | --- | --- | --- | --- | --- | --- |
| *Self reported symptoms at screening* | | |  |  |  |  |
| Vaginal discharge reported: | |  |  |  |  |  |
|  | No | 18 (8) | 135 (60) | 50 (22.2) | 22 (9.8) | 0.08 |
|  | Yes | 1 (20) | 1 (20) | 3 (60) | 0 (0) |  |
| *Clinician observed findings at screening* | | |  |  |  |  |
| Cervical mucus^a^: | |  |  |  |  |  |
|  | No | 5 (3.8) | 82 (62.6) | 31 (23.7) | 13 (9.9) | 0.09 |
|  | Mild | 11 (12.6) | 48 (55.2) | 19 (21.8) | 9 (10.3) |  |
|  | Abundant | 2 (28.6) | 3 (42.9) | 2 (28.6) | 0 (0) |  |
| Cervical and/or vaginal epithelial abnormalities: | | |  |  |  |  |
|  | No | 15 (7.2) | 124 (59.6) | 48 (23.1) | 21 (10.1) | 0.34 |
|  | Yes | 4 (18.2) | 12 (54.5) | 5 (22.7) | 1 (4.5) | 0.34 |
| Ectopy: | |  |  |  |  |  |
|  | No | 11 (8.4) | 73 (55.7) | 33 (25.2) | 14 (10.7) | 0.66 |
|  | Yes | 8 (8.1) | 63 (63.6) | 20 (20.2) | 8 (8.1) |  |
| Any colposcopy finding?: | |  |  |  |  |  |
|  | No | 17 (8.5) | 119 (59.8) | 45 (22.6) | 18 (9) | 0.83 |
|  | Yes | 2 (6.5) | 17 (54.8) | 8 (25.8) | 4 (12.9) |  |
| *Laboratory-confirmed reproductive and urinary tract infections* | | | | |  |  |
| HIV serology: | |  |  |  |  |  |
|  | Negative | 19 (8.7) | 128 (58.4) | 50 (22.8) | 22 (10) | 0.75 |
|  | Positive | 0 (0) | 7 (70) | 3 (30) | 0 (0) |  |
| HSV-2 serology: | |  |  |  |  |  |
|  | Negative | 15 (10.3) | 85 (58.2) | 33 (22.6) | 13 (8.9) | 0.54 |
|  | Positive | 4 (4.8) | 51 (60.7) | 20 (23.8) | 9 (10.7) |  |
| Bacterial STI^b^ | |  |  |  |  |  |
|  | Negative | 17 (8.9) | 116 (60.4) | 41 (21.4) | 18 (9.4) | 0.35 |
|  | Positive | 1 (2.7) | 20 (54.1) | 12 (32.4) | 4 (10.8) |  |
| BV by Nugent score: | |  |  |  |  |  |
|  | 0-3 | 15 (13.3) | 93 (82.3) | 2 (1.8) | 3 (2.7) | < 0.001 |
|  | 4 - 6 | 1 (5.0) | 15 (75.0) | 3 (15.0) | 1 (5.0) |  |
|  | 7 - 10 | 0 (0.0) | 14 (18.7) | 43 (57.3) | 18 (24.0) |  |
| BV by Amsel criteria: | |  |  |  |  |  |
|  | Negative | 18 (9.1) | 128 (65.0) | 37 (18.8) | 14 (7.1) | < 0.001 |
|  | Positive | 1 (3.0) | 8 (24.2) | 16 (48.5) | 8 (24.2) |  |
| Candidiasis on wet mount: | |  |  |  |  |  |
|  | Negative | 16 (8) | 113 (56.5) | 50 (25) | 21 (10.5) | 0.12 |
|  | Positive | 3 (10) | 23 (76.7) | 3 (10) | 1 (3.3) |  |
| Urinary tract infection by dipstick test: | | |  |  |  |  |
|  | Negative | 4 (2.6) | 4 (2.6) | 132 (85.7) | 14 (9.1) | 0.10 |
|  | Positive | 0 (0.0) | 5 (9.4) | 41 (77.4) | 7 (13.2) |  |
| *Treatment in the 14 days prior to enrollment* | | |  |  |  |  |
| Any systemic antibiotics: | |  |  |  |  |  |
|  | No | 18 (9.4) | 115 (59.9) | 39 (20.3) | 20 (10.4) | 0.12 |
|  | Yes | 1 (2.6) | 21 (55.3) | 14 (36.8) | 2 (5.3) |  |
| Any vaginal antibiotics: | |  |  |  |  |  |
|  | No | 19 (8.3) | 136 (59.6) | 51 (22.4) | 22 (9.6) | 0.17 |
|  | Yes | 0 (0) | 0 (0) | 2 (100) | 0 (0) |  |
| Bacterial vaginosis requiring treatment: | | |  |  |  |  |
|  | No | 19 (8.7) | 130 (59.6) | 47 (21.6) | 22 (10.1) | 0.17 |
|  | Yes | 0 (0) | 6 (50) | 6 (50) | 0 (0) |  |
| Candidiasis requiring treatment: | |  |  |  |  |  |
|  | No | 17 (8.5) | 116 (58) | 46 (23) | 21 (10.5) | 0.11 |
|  | Yes | 0 (0) | 10 (100) | 0 (0) | 0 (0) |  |

An expanded version of Table 2 in the manuscript, showing clinical characteristics of women in the three VMB clusters but also in the women not assigned to a cluster. "KRST" represents the first letter of the names of the four African countries where the study was conducted (Kenya, Rwanda, South Africa and Tanzania) and "KRST-pIII-V" denotes a new KRST cluster formed by collapsing KRST-III, KRST-IV and KRST-V. "UA" stands for unassigned.

^*^Fisher’s exact test in bivariable analysis

^a^4/230 values missing

^b^Bacterial STIs includes syphilis (by serology), chlamydia and gonorrhea (by PCR), and trichomoniasis (by InPouch culture test).
